# Novel insights on targeting ferroptosis in cancer therapy

**DOI:** 10.1186/s40364-020-00229-w

**Published:** 2020-10-02

**Authors:** Sipeng Zuo, Jie Yu, Hui Pan, Linna Lu

**Affiliations:** 1 Department of Ophthalmology, Ninth People’s Hospital, Shanghai Jiao Tong University School of Medicine, Shanghai, P. R. China; 2 Shanghai Key Laboratory of Orbital Diseases and Ocular Oncology, No. 12, Lane 833, Zhizaoju Road, Huangpu District, Shanghai, 200001 P. R. China

**Keywords:** Ferroptosis, Cancer, Therapy, Signaling pathway

## Abstract

Ferroptosis belongs to a novel form of regulated cell death. It is characterized by iron dependence, destruction of intracellular redox balance and non-apoptosis. And cellular structure and molecules level changes also occur abnormally during ferroptosis. It has been proved that ferroptosis exist widespreadly in many diseases, such as heart disease, brain damage or alzheimer disease. At the same time, the role of ferroptosis in cancer cannot be underestimated. More and more indications have told that ferroptosis is becoming a powerful weapon against cancer. In addition, therapies rely on ferroptosis have been applied to the clinic. Therefore, it is necessary to understand this newly discovered form of cell death and its connection with cancer. This review summarizes the mechanism of ferroptosis, ferroptosis inducers based on different targets and inspection methods. At last, we analyzed the relationship between ferroptosis and malignancies, in order to provide a novel theory basis for cancer treatment.

## Introduction

Death is the ultimate destination of cells. The various forms of cell death are apoptosis, necrosis, pyroptosis, oncosis, and autophagy, and each form has its respective features [1]. A disruption of cell death as an approach to treat tumors has been pursued by numerous researchers. As a controlled cellular process, cell death is essential for maintaining tissue integrity and homeostasis, so a disruption of this process under human intervention may show clinical effects [2].

Ferroptosis, another form of cell death, has been observed many times. Cell death caused by insufficient cystine was discovered in the 1950s, and it could be rescued by the lipophilic antioxidant, tocopherol, a component of vitamin E [3, 4]. Subsequently, lipid peroxidation and reactive oxygen species (ROS) were found to be unfavorable for ferroptosis, and another key enzyme, glutathione peroxidase 4 (GPX4), was isolated, purified, and confirmed to be involved in the cellular response to oxidative stress [5–7]. Interestingly, GPX4 overexpression has been reported to prevent oxidative stress-induced death in a mouse model [8]. The term ferroptosis, which referred to a novel type of iron-dependent programmed cell death, was introduced in 2012 [9]. Ferroptosis differs from other forms of cell death at biochemical, morphological, and genetic levels. Ferroptosis is characterized by its iron dependence, ability to disrupt the intracellular redox balance, and absence of apoptosis [10]. The differences between ferroptosis and apoptosis lie first in morphological features. When ferroptosis occurs, cell cytoplasm presents round and detached, mitochondrial membrane densities condense, mitochondria crista reduces or vanishes, and outer mitochondrial membrane ruptures [11]. When apoptosis occurs, plasma membrane bubbles, pseudopods retract, and cellular volume reduces, nuclear fragments, chromatin condenses [11, 12]. In addition, ferroptosis generally is pro-inflammatory owing to release of damage-associated molecular pattern molecules and apoptosis is often anti-inflammatory and immune-silent except in some cases with activation of caspases and oligonucleosomal DNA fragmentation. Besides, not the same core regulators play parts in apoptosis and ferroptosis [11]. Studies have demonstrated that ferroptosis is the result of lipid peroxidation, and molecules that directly or indirectly regulate iron metabolism and lipid peroxidation can induce or inhibit ferroptosis. These molecules are mitochondrial voltage-dependent anion channels 2/3 (VDAC2/3), GPX4, ferroptosis suppressor protein 1, heat shock protein (HSP), nuclear factor erythroid 2-related factor 2 (NRF2), nicotinamide adenine dinucleotide phosphate (NADPH) oxidase (NOX), and p53 [11, 13–17]. While some other molecules (p53, Bcl-2 family proteins) are core regulators of apoptosis.

Interestingly, emerging studies have already reported that ferroptosis is related tightly with autophagy [18]. Autophagy refer to a conservative catabolic process that transfer cellular components to lysosomes for degradation [19]. During occurrence of ferroptosis, after cystine deprivation (one of ferroptosis induction condition), which activated autophagy, the iron storage protein ferritin is degraded, which is mediated by the cargo receptor NCOA4. Degraded ferritin results in more Fe releasing, which launch ferroptosis subsequently [18]. In addition, total ROS level of autophagy-deficient cells is significantly lower than autophagy-rescued cells. Therefore, autophagy is required for ROS accumulation in ferroptosis [18]. Because of autophagic specific connection with ferroptosis, drug repositioning therapeutic strategies targeting autophagy to induce cancer cell death are promising [20, 21].

Researchers have reported that ferroptosis can be induced experimentally by various molecules such as the classic inducer, erastin. In addition, ferroptosis has been observed in several types of cancer; therefore, disrupting ferroptosis may offer new treatment options. Here, we discuss the mechanism of action of ferroptosis, describe different ferroptosis inducers according to their targets, and discuss inspection methods. Lastly, the clinical significance of ferroptosis in cancer is presented.

### Mechanism of ferroptosis

**Fig. 1:**
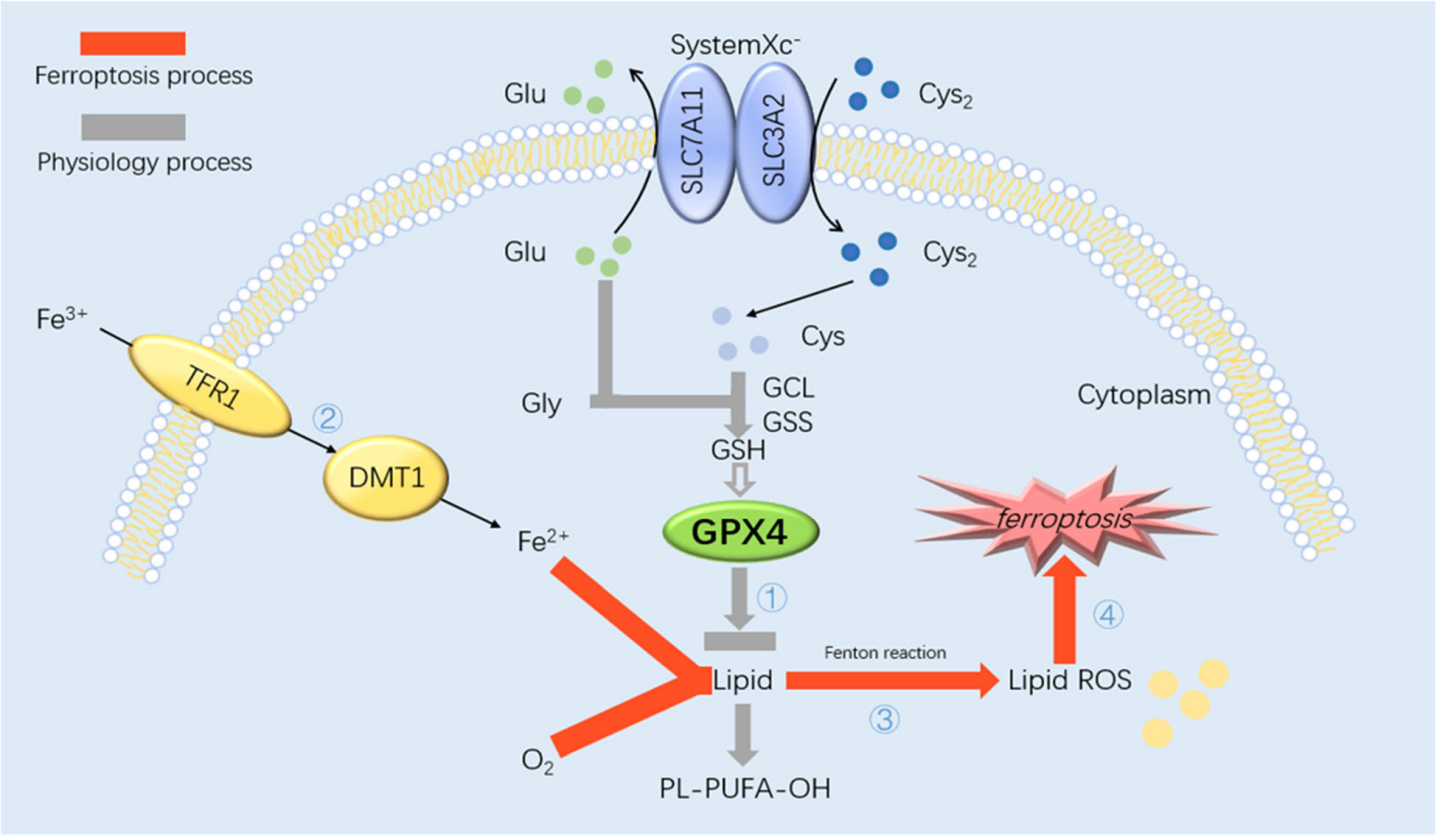
Sketch of ferroptosis mechanism. The mechanism of ferroptosis in cells. Cys_2_ that enter the cytoplasm via systemXc^−^ synthesize GSH with glutamate, glycine and cysteine. GPX4 is a kind of GSH-dependent reductase. When GPX4 dysfunction, lipid transforms into lipid ROS with O_2_ and Fe^2+^. L-ROS attack intracellular biomolecules and kill the cell

SystemXc^−^, a heterodimeric antiporter with important roles in the metabolism of membrane lipids, binds to the twelve-pass transmembrane transporter protein SLC7A11 (xCT) and the single-pass transmembrane regulatory protein SLC3A2 (CD98) via disulfide bonds. Cystine and glutamic acid enter cells through systemXc^−^ on cell membranes. And then the following procedure is the synthesis of glutathione (GSH) as the substrate of GPX4 through γ-glutamyl cysteine ligase and GSH synthase [22–24]. In general, membrane lipid metabolism is accomplished by the reductase GPX4 and the existence of GSH guarantee GPX4 normal physiological function. A previous study has reported that GPX4, as a key reductase in the pathogenesis of ferroptosis, can mediate the breakdown of membrane lipids [25]. This is the first step involved in ferroptosis to make GPX4 inactivated. Secondly, Iron (Fe) enters cells by binding to the transferrin receptor 1 (TFR1) on cell membranes and then ferrous ions are transported into the cytosol by the divalent metal ion transporter 1. During ferroptosis, membrane lipids bypass the GPX4 pathway instead, they are oxidized into lipid ROS via the Fenton reaction, with electrons provided by ferrous ions, upon some specific induction condition such as cystine deprivation or excess Fe ions. The Fenton reaction is one of the main ways in which ferroptosis occurs [26]. At last, it is the way in which iron and hydrogen peroxide oxidize various substrates and cause cellular damage [27]. Under conditions of low oxygen, lipid ROS attack important intracellular biomolecules, such as DNA, RNA, and proteins, which disrupts the cellular homeostasis and causes the irreversible death of cells [11, 12, 28], as shown in Fig. 1. And relevant biomarkers are listed in Table 1.

**Table 1:** Biomarkers for ferroptosis

Core molecules markers	Biochemistry	Morphology
①Pro-ferroptosis: TFR1 VDAC2/3 Ras P53 NOX ②Anti-ferroptosis: SLC7A11 HSPB1 NRF2 GSH GPX4	①Activation of MAPKs ②Iron and ROS accumulation ③Inhibition of system Xc^−^ with decreased cystine uptake ④GSH depletion ⑤Increased NAPDH oxidation	①Round and detached cell cytoplasm ②Condensed mitochondrial membrane densities ③Reduced or vanishing mitochondria crista ④Outer mitochondrial membrane rupture

### Ferroptosis inducers

Ferroptosis is characterized by systemXc^−^, GPX4, ferrous ions and other biomolecules. Small exogenous molecules, changes in the cellular environment, changes in the activity/function of key biomolecules, abnormal levels of key cellular molecules, and metabolic disturbances can affect ferroptosis. Ferroptosis inducers can be divided into two classes, namely those that target systemXc^−^ (class 1) and those that inhibit GPX4 activity (class 2). In addition, ferroptosis can be induced by other inducers (Table 2) and high or low concentrations of intra- or extracellular molecules such as glutamic acid (Glu) or Fe. Clinical studies have reported that classical ferroptosis inducers can inhibit cancer progression and prolong the survival time of patients. Ferroptosis inducers have been used in the synthesis of drug-encased nanoparticles to induce ferroptosis in cancer cells. In the subsequent sections, we summarize the different types of ferroptosis inducers and discuss their mechanisms of action in cancer.

**Table 2:** Ferroptosis inducers

Category	Molecule	Target
SystemXc^−^ Inhibition	Erastin	SystemXc^−^/VDAC/TFR1
	SAS	SystemXc^−^
	SRF	SystemXc^−^
GPX4 Inhibition	RSL3/5	GPX4/VDAC
	FINs	GPX4
	FINO2	GPX4
	DPIs	GPX4
	Altretamine	GPX4
	Withaferin A	GPX4
Others classes	GLU	Cystine
	Iron carrier	Iron
	BSO	GSH
	DPI2	GSH
	Cisplatin	GSH
	Artemisinin and derivative	Iron
	Nanoparticle inducers	

### Inhibition of systemXc^−^

SystemXc^−^ has important roles in the transport of amino acids during ferroptosis, suggesting that cancer cells rely on systemXc^−^. SystemXc^−^ inhibitors can inhibit cystine uptake and disrupt protein folding, thereby inducing ferroptosis.

#### Erastin and derivatives

Erastin, a small molecule compound discovered by Dolma and colleagues in 2003, selectively kills RAS-expressing cancer cells [29]. Molecular mechanism studies have revealed that mutant *RAS* can significantly promote the expression of transcription factor NRF2 and further activate the downstream important factor, SLC7A11 [30]. Therefore, erastin is a ferroptosis inducer that down-regulates the GSH level by inhibiting systemXc^−^, which is caused by the unique structural features of erastin. Erastin also targets VDAC2/3, a mitochondrial membrane exchange channel, and regulates its permeability, causing mitochondrial disorders, production of ROS, and ferroptosis [31, 32]. Furthermore, another study has demonstrated that erastin and its derivatives, such as piperazine erastin, show inducing effectiveness [12]. Additionally, erastin can also affect the breakdown of iron by inhibiting TFR1 [33], and the ferroptosis inhibitor, HSPB1, can reduce the intracellular iron concentration by inhibiting the expression of TFR1. Therefore, HSPB1 overexpression can inhibit ferroptosis [34].

#### Others

The effects of Salazosulfapyridine and sorafenib are similar to those of erastin. The United States Food and Drug Administration has approved Salazosulfapyridine for the treatment of rheumatoid arthritis. Similar to erastin, Salazosulfapyridine can induce ferroptosis by suppressing systemXc^−^, although the efficacy of Salazosulfapyridine is weaker than that of erastin [35]. Nevertheless, Salazosulfapyridine can enhance the efficacy of other chemotherapeutic drugs that target glioma [36]. By contrast, the concrete mechanism of action of sorafenib remains to be studied, although two approaches have been proposed [37].

### GPX4 inhibition and degradation

SystemXc^−^ inhibitors induce ferroptosis by affecting the function of systemXc^−^. However, some cancer cells can synthesize cysteine and methionine through amino acid metabolic pathways, and systemXc^−^ inhibitors have no effect on these cells. GPX4, as a GSH-dependent enzyme, can suppress the production of lipid ROS. Furthermore, GPX4 is overexpressed in most cancer cells, and the manipulation of GPX4 function can affect the extent of ferroptosis in cancer cells [38].

#### RSL3/5

RSL refers to RAS Selective Lethal small molecules, which were isolated by the Stockwell laboratory in 2008 [39]. The chloroacetamide moiety of RSL3 is essential for GPX4 function. Specifically, RSL3 combines with GPX4 through the alkylation of the selenocysteine, thereby resulting in the inactivation of GPX4. Furthermore, caspase inhibitors could not reverse RSL3-induced cell death, suggesting that this type of cell death is not caspase-dependent. By contrast, RSL5 requires VDAC2/3, as well as protein synthesis, to induce ferroptosis [39].

#### FINs

Category II FINS were identified during a large screening of potential ferroptosis inducers. FIN56 (Mr 517.66), a typical FIN derived from CIL56, inhibits the activity of GPX4 without reducing the GSH level. Compared to CIL56, FIN56 is a more potent inducer of ferroptosis [40, 41]. FIN56 induces ferroptosis through two mechanisms. It can promote the degradation of GPX4 with the aid of acetyl-CoA carboxylase or activate squalene synthase to deplete the antioxidant coenzyme Q10. The oxime moiety in FIN56 is critical for ferroptosis, and the hydrophobicity of the piperidine determines if ferroptosis will be induced [42, 43].

#### Others

FINO2 is an organic peroxide with a mechanism of action similar to that of artemisinin; however, only the portion of the molecule containing 1,2-dioxolane has been reported to induce ferroptosis. Similar to RSL3, FINO2-induced ferroptosis is not dependent on the caspase pathway; instead, FINO2 deactivates GPX4 and oxidizes Fe directly [37]. Due to the high levels of Fe in cancer cells, ferroptosis is easily induced in cancer cells by FINO2, and its endo-peroxide structure and polar head are largely responsible for this induction. Other small molecules can also inhibit GXP4 through different mechanisms. DPI7 (ML162), DPI10 (ML210), DPI12, DPI13, DPI17–19 inhibit GPX4 by covalent binding, altretamine inhibits GPX4 directly, and withaferin A inactivates GPX4 by increasing the Fe^2+^ level, and thereby inducing ferroptosis [44].

### Other classes

#### Glutamic acid

Ferroptosis has been observed in brains exposed to large amounts of Glu, which may have inhibited the function of systemXc^−^ [45]. Ferroptosis is also involved in ischemia–reperfusion injury in the heart and kidneys under presence of Glu [46, 47].

#### Iron carriers

Except for glutamate, superfluous Fe ions can induce ferroptosis, and the modality of iron loading involves various malysites, such as ferrous chloride, ferrous ammonium sulfate, ferric ammonium citrate, and haemin/hemoglobin. Superfluous Fe ions have been reported to induce lipid peroxidation, ROS accumulation, and cell death [48–50].

#### GSH synthesis inhibitors

In the absence of GSH, the function of GPX4 is compromised and the generation of ROS is increased by the Fenton reaction. Buthionine sulfoximine is an inhibitor of GSH synthesis. A low GSH level can also hinder ferroptosis. Both DPI2 and cisplatin, as inhibitors of GSH synthesis, can induce ferroptosis by depleting GSH from cells [42].

#### Artemisinin and its derivatives

Artemisinin, known for its treatment of malaria, can interfere with the redox balance in cells; therefore, the cytotoxic effects of this compound are closely related to those of Fe ions. Pancreatic ductal adenocarcinoma cells rarely undergo apoptosis, but they can be killed by artesunate-induced ferroptosis. In addition, the artemisinin derivative, DHA, is effective against acute myeloid leukemia, as well as head and neck squamous cell carcinoma, where it has been shown to degrade ferritin by autophagy [12, 51].

#### Nanoparticle inducers

Nanomaterials are believed to induce ferroptosis by participating in diverse biochemical reactions and interfering with the metabolic balance of cells. In 2016, Kim and colleagues first reported that nanoparticles can induce ferroptosis. They induced ferroptosis in cancer cells by structuring α-MSH-PEG-C′ dots with near-infrared fluorescent ultra-small silica nanoparticles (C′ dots, ~ 6 nm), polyethylene glycol (pegylated C′ dots), and alpha-melanocyte stimulating hormone (α-MSH) and reported an increase in the Fe level, an accumulation of ROS, and a decrease in the GSH level [52, 53].

After that, nanoparticle inducers facilitate ferroptosis using a mechanism similar to that of previously mentioned molecules. It’s been known that the natural omega 3 fatty acid docosahexaenoic acid depletes GSH, inactivates GPX4, and increases ROS levels [54, 55]. By contrast, SRF@Fe^III^TA (made up of Fe^3+^ ion, tannic acid and sorafenib), MON-p53 (metal-organic network encapsulated with p53 plasmid), DGU:Fe/Dox (a nanolongan delivery system releasing Fe^3+^ and doxorubicin), and PEGylated single-atom Fe-containing nanocatalysts can increase intracellular Fe^2+^ levels [56–59]. Studies are underway to identify more potent nanoparticles with fewer side effects.

### Methods of measurement

Ferroptosis can be observed and measured by transmission electron microscopy, and the results of these morphological and biochemical studies have revealed that ferroptosis can induce diverse cellular changes, indicating that it affects different molecules. A previous study reported that ferroptotic cells are round and detached, with mitochondrial damage (i.e., shrinkage or loss of cristae, fragmentation of outer membrane); however, nuclei remain intact [12].ROS accumulation is the most important predictor of ferroptosis. ROS levels can be measured by fluorescent sensors, such as 2′,7′-dichlorodihydrofluorescein diacetate and MitoSOX, as well as other probes such as LiperFluo [60] and isoprostane [61]. For instance, MitoSOX is used to measure ROS levels in mitochondria [62].

GPX4 participates in the breakdown of membrane lipids. It also has important roles in the prevention of cell death. GPX4 activity can be measured by various approaches. In the first approach, the NADPH oxidation rate, which correlates with the reduction of tertbutylhydroperoxide by GPX4, is measured. In the second approach, the phosphatidylcholine hydroperoxide level is quantified by liquid chromatography–mass spectrometry or immunoblot analysis. The level of prominin 2, a stress response protein, can also be measured [63, 64].

As previously discussed, Fe can promote ferroptosis. However, there is no method to measure the Fe level in viable cells. Furthermore, it is not useful to measure the total Fe concentration because redox reactions do not require Fe atoms; instead, methods that measure the ferrous ion concentration should be used. RhoNox-1, the first activated fluorescent probe, utilizes the ferrous ion-mediated deoxygenation of the tertiary amine N-oxide to determine the ferrous ion concentration in real-time. As such, RhoNox-1 has overcome the disadvantages of chelation-based probes in the measurement of ROS levels [65]. Moreover, FRET iron probe 1, an endoperoxide reactivity-based Förster resonance energy transfer probe, can measure the labile Fe pool in viable cells by destroying endoperoxide bridges in the Cy3 triplet molecule [66]. By contrast, the binding of chelation-based probes, such as Phen Green and PRA, to substrates is weaker and less selective in the measurement of Fe levels in cells.

### Ferroptosis and carcinoma

**Fig. 2:**
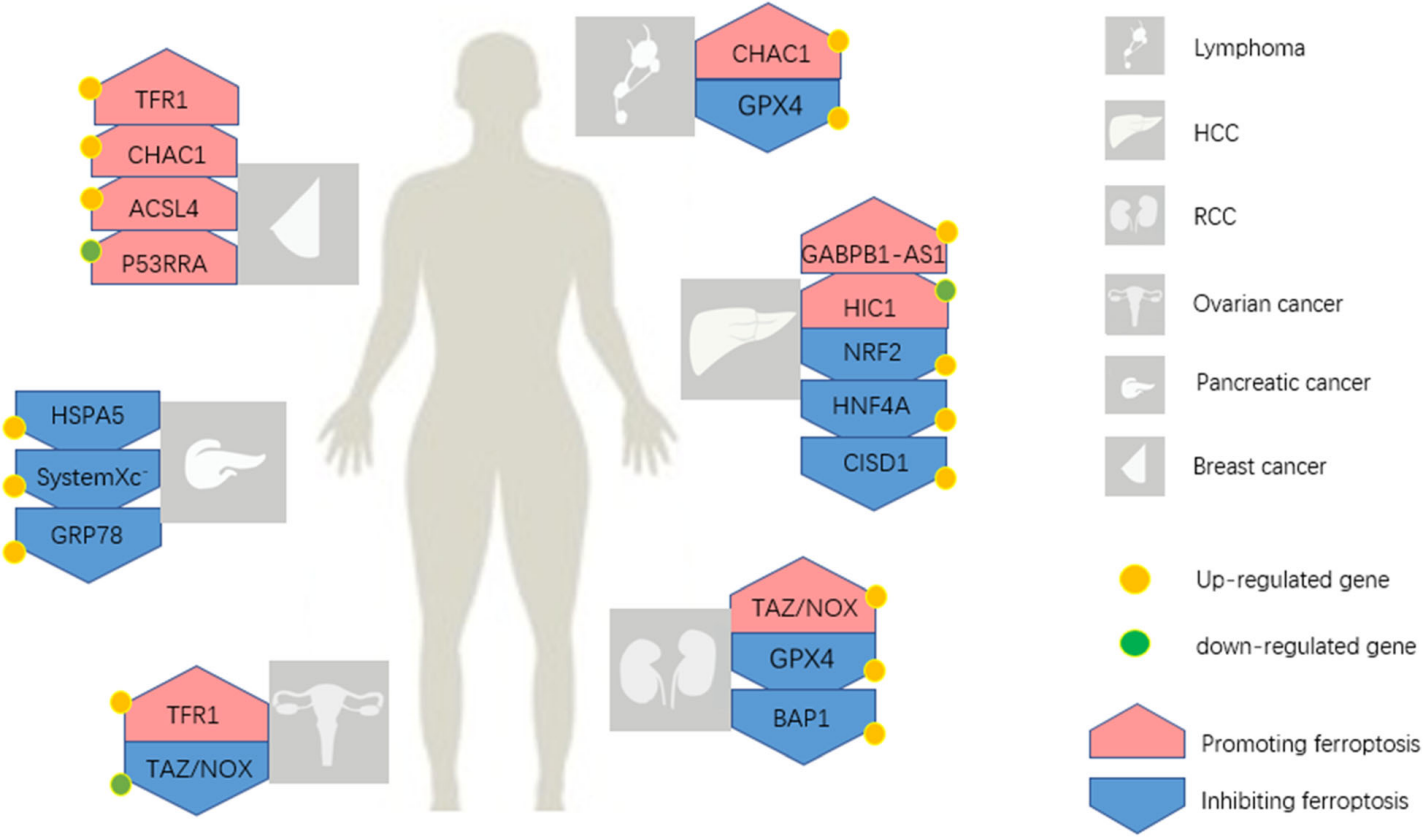
Known genetic effects on various cancers via ferroptosis. The known genetic effects via ferroptosis in dominating cancers. These cancers include lymphoma, HCC, RCC, ovarian cancer, pancreatic cancer, and breast cancer. They have different sensitivities to ferroptosis

Research on the significance of ferroptosis in cancer has recently gained momentum. Cancer cells are more susceptible to ferroptosis due to high cellular activity (Table 3). For instance, the HSP level is elevated in cancer cells, and HSP can inhibit ferroptosis in these cells [85], suggesting that ferroptosis may offer new treatment options. Recent main advancements in the field are summarized in Fig. 2.

**Table 3:** Known genes effects on ferroptosis in various cancers

Tumor type	Gene	Effect	References	Mechanism
Lymphoma	*GPX4*	Inhibition	Yang, W.S et al. [35]	Reduce the accumulation of ROS
	*CHAC1*	Promotion	Wang et al. [65]	Degrade GSH
HCC	*NRF2*	Inhibition	Sun, X et al. [67]	Encode antioxidant proteins
	*HIC1*	Promotion	Xiao Zhang et al. [68]	Affect the synthesis of GSH
	*HNF4A*	Inhibition		
	*CISD1*	Inhibition	Yuan, H et al. [69]	Suppress mitochondrial lipid peroxidation
	*GABPB1-AS1*	Promotion	Qi, W et al. [70]	Inhibited the cellular antioxidant capacity
RCC	*GPX4*	Inhibition	Yang et al. [35]	Reduce the accumulation of ROS
	*TAZ/NOX*	Promotion	Yang et al. [71]	Exhaust intracellular NADPH
	*BAP1*	Inhibition	Zhang, Y et al. [14]	Decreases SLC7A11 expression
Ovarian cancer	*TFR1*	Promotion	Pizzamiglio et al. [72]	Increase the intake of iron
	*TAZ/NOX*	Inhibition	Yang et al. [73]	Reduce the depletion of NADPH
Pancreatic cancer	*HSPA5*	Inhibition	Zhu, S et al. [74]	Inhibit the expression of TFR1
	*SystemXc*^*−*^	Inhibition	Lo M et al. [75]	Increase the synthesis of GSH
	*GRP78*	Inhibition	Wang, K et al. [76]	Unkown
Breast cancer	*TFR1*	Promotion	Basuli et al. [77]	Increase the intake of iron
	*CHAC1*	Promotion	Chen et al. [78]	Degrade GSH
	*ACSL4*	Promotion	Doll, S et al. [79]	Increasing cellular lipid composition
	*P53RRA*	Promotion	Chao Mao et al. [80]	Nuclear sequestration of p53
Head and neck cancer	*NRF2*	Inhibition	Sun, X et al. [81]	Encode antioxidant proteins
Colorectal cancer	*P53*	Inhibition	Xie Y et al. [82]	Increase the expression of systemXc^−^
Cervical carcinoma Melanoma	*HSPB1* *SLC1A5* *GOT1*	Inhibition Promotion Inhibition	Stuart et al. [62] Sun, X et al. [29] Luo, M et al. [83] Zhang, K et al. [84]	Inhibit the expression of TFR1 Reduce the accumulation of Glu Decrease the depletion of Glu

### Ferroptosis and lymphoma

Diffuse large B cell lymphoma (DLBCL) is the most common type of non-Hodgkin’s lymphoma, accounting for approximately 30–40% of all cases [86]. DLBCL is sensitive to GPX4-induced ferroptosis [42], and GPX4 expression is negatively correlated with the prognosis. Thus, GPX4 is a promising prognostic predictor [87]. As for Burkitt’s lymphoma, Wang and colleagues confirmed that artesunate can activate the ATF4–CHOP–CHAC1 pathway, thereby inhibiting cancer cell proliferation and inducing ferroptosis [67]. In addition, *ALOX12* can promote ferroptosis in *p53*-dependent cancers without affecting GPX4 function [88]. Deletion of the *ALOX12* allele can accelerate tumorigenesis in an Eμ-myc lymphoma model, suggesting that it may be a potential therapeutic target for *p53*-dependent tumors.

### Ferroptosis and hepatocellular cancer

Hepatocellular carcinoma is often diagnosed at late stages, and it is ranked as the third cause of mortality in males worldwide [89]. Sorafenib, a non-specific kinase inhibitor, is used in the treatment of advanced hepatocellular carcinoma, prolonging the survival of patients by several months. Sorafenib can induce ferroptosis in hepatic cancer cells independent of its kinase inhibitory effect [24, 90], although drug resistance is a common manifestation of treatment. Drug resistance is caused by the activation of the p62–Keap1–NRF2 pathway. The degradation of NRF2 is inhibited by the binding of p62 to Keap1, which leads to the accumulation of NRF2 in cells. NRF2 can up-regulate diverse ferroptosis inhibitors, such as NQO1 and FTH1, and sensitize hepatic cancer cells to sorafenib and erastin both in vitro and in vivo. Furthermore, high NRF2 levels in cells can promote the transcription of genes that encode antioxidant proteins, thereby protecting hepatic cancer cells from ferroptosis [68].

Retinoblastoma protein (Rb) is an important regulator of cell proliferation [69], and gene mutations can promote hepatocellular carcinoma. Ferroptosis has been observed in sorafenib-treated hepatic cancer cells with negative Rb expression, with a two- to three-fold increase in cell death [70]. Moreover, the resistance of hepatic cancer cells to sorafenib has also been reported to involve metallothionein-1G [91] and sigma 1 receptor [92]. Zhang and colleagues studied two transcription factors, namely HIC1 and HNF4A, and reported that decreased HIC1 expression promotes ferroptosis, whereas increased HNF4A expression inhibits ferroptosis [71]. In addition, CDGSH iron sulfur domain 1 overexpression in human hepatic cancer cell lines, such as HepG2 and Hep3B, can inhibit mitochondrial lipid peroxidation and ferroptosis [93].

Long non-coding RNAs (LncRNAs) have important roles in ferroptosis. For instance, lncRNA GABPB1-AS1 can down-regulate the GABPB1 level and inhibit its antioxidant capacity, thereby inducing ferroptosis in hepatic cancer cells [77].

### Ferroptosis and renal cell cancer

Clear cell renal cell carcinoma (CCRCC) is the most prevalent type of kidney cancer. CCRCC cells lacking GPX4 expression are susceptible to death because of ROS accumulation [94]. Hereditary leiomyomatosis and renal cell cancer is a rare disease caused by the inactivation of fumarate hydratase in the Krebs cycle, which results in the accumulation of fumarates and the alteration of GPX4 function, thereby increasing the sensitivity of cancer cells to ferroptosis [73].

RCC cells express density-dependent TAZ, an effector in the Hippo–YAP/TAZ pathway, which are generally regarded as oncogene highly conserved in evolution. TAZ can promote the expression of epithelial membrane protein 1 and NOX4, which accelerate the kinetics of ferroptosis by increasing the accumulation of ROS [95]. In addition, it’s been found that in renal cancer cell line, as a nuclear deubiquitinating enzyme, the tumour suppressor BRCA1-associated protein 1 decreases SLC7A11 expression, thereby causing lipid peroxidation and ferroptosis [16].

### Ferroptosis and ovarian cancer

Similar to hepatocellular carcinoma, ovarian cancer is often diagnosed at late stages [96]. Ferroptosis can enhance the chemosensitivity of ovarian cancer cells. For instance, serous ovarian cancer cells overexpressing TFR1 are sensitive to ferroptosis [97], whereas ovarian cancer tumor-initiating cells and ovarian cancer cells are sensitive to ferroptosis inducers [97]. Erastin can reverse the resistance of ovarian cancer cells to docetaxel [98]. Similar to renal cell carcinoma, the TAZ/NOX pathway mediates ferroptosis and chemoresistance in ovarian cancer cells, and low TAZ expression in patients with recurrent ovarian cancer is responsible for the reduced susceptibility to ferroptosis [74].

Artensunate, as a derivative of artemisinin and an inducer of ferroptosis, can inhibit the proliferation of primary ovarian cancer cells and ovarian cancer cell lines [99]. Meanwhile, the mechanism of action of ferroptosis also involves the enzyme, stearoyl-CoA desaturase 1 (SCD1), which stimulates fatty acid synthesis. If SCD1 is inhibited, then the anti-tumor effects of ferroptosis will be enhanced. Therefore, the combined use of an SCD1 inhibitor and a ferroptosis inducer may represent a new therapeutic approach [75, 100].

### Ferroptosis and pancreatic cancer

Pancreatic cancer is a highly lethal disease [101]. HSPA5, a member of the HSP70 family, negatively regulates ferroptosis in human pancreatic ductal adenocarcinoma cells [76]. Furthermore, a previous study reported that artensunate can induce ferroptosis in pancreatic ductal adenocarcinoma cells expressing constitutively active *KRas*, although normal cells were not affected [51]. In addition, systemXc^−^ has important roles in pancreatic cancer and drug resistance because of its function in the transport of cystine, and dysfunction of systemXc^−^ has curative effect on pancreatic cancer [72, 102, 103]. Finally, glucose-regulated protein 78 (GRP78) is a molecular chaperone, and the activation of GRP78 can suppress ferroptosis in pancreatic cancer caused by a *KRAS* mutation [78].

### Ferroptosis and breast cancer

Breast cancer is one of the most prevalent cancers in women worldwide and has witnessed an improvement in its prognosis due to advancements in the field [79]. The elevated expression of TFR1 in breast cancer cells renders them susceptible to ferroptosis, which may represent a new therapeutic approach [80]. It’s reported that siramesine and lapatinib are effective ferroptosis inducers in breast cancer [104]. However, an elevated estrogen level can reduce sulfasalazine-induced ferroptosis through the transferrin receptor [81], suggesting that hormones affect ferroptosis.

CHAC1 as a downstream protein of activator of transcription factor 4 (ATF4) can degrade GSH [105]. CHAC1 induces ferroptosis in human triple negative breast cancer cells via the GCN2–eIF2α–ATF4 pathway [106]. Except that, acyl-CoA synthetase long-chain family member 4 can promote ferroptosis by increasing the content of intracellular lipids [82]. Another study reported that the down-regulation of lncRNA P53RRA promoted ferroptosis by sequestering p53 in the nucleus [83]. In addition, as a herb widely used in traditional Chinese medicine, danshen has been reported to improve the prognosis of breast cancer, which may be due to the effects of its bioactive compound, dihydroisotanshinone I, in repressing the protein GPX4 expression and inducing ferroptosis [84].

### Ferroptosis and other cancer

Ferroptosis has also been observed in other types of cancers such as prostate and cervical cancer [34]. Head and neck cancer expressing a low level of NRF2 is sensitive to drug-induced ferroptosis. Therefore, the activation of the NRF2–ARE pathway protects cells from the effects of ferroptosis inducers [107, 108]. Mutations in *p53* also protect colorectal cancer cells from the effects of erastin-induced ferroptosis [109], which may offer a new treatment option. Moreover, ferroptosis is uncommon in cervical carcinoma cells highly expressing HSPB1 according to recent reports [34, 85], suggesting HSPB1 provide protective effects on cancer cells.

In melanoma, decreased SLC1A5 expression has been reported to reduce glutamine accumulation, decrease Glu production, and increase ferroptosis [110]. In addition, the decrease in glutamic-oxaloacetic transaminase prevented the depletion of Glu, thereby resulting in anti-ferroptosis of melanoma cells [111].

In gastrointestinal cancer, study claimed that CD44v, the variant of CD44 (an adhesion molecule overexpressed in cancer stem-like cells), can interact with the xCT, thereby stabilizing the intracellular level of GSH and defensing against ferroptosis [112]. Given that sulfasalazine (SSZ) inhibits xCT, it is highly likely that SSZ induces ferroptosis of cancer stem-like cells. Therefore, therapeutic strategies that target inducing ferroptosis of cancer stem cells through the molecules localized by xCT may be pretty promising [20, 113].

## Conclusions

Ferroptosis, a novel form of cell death, is characterized by iron dependence, disruption of the intracellular redox balance, and absence of apoptosis. The application of ferroptosis in the treatment of cancer has recently gained momentum, as the process can inhibit tumor growth and improve the efficacy of chemotherapeutic drugs.

Fe, a trace element, is present in high concentrations in the human body. Interestingly, its concentration is higher in cancer cells than that in normal cells. Presently, chemotherapeutic drugs are widely used to kill cancer cells. However, cancer cells can develop resistance to drugs, leading to poor prognosis and low survival rates. Studies have reported an association between ferroptosis and drug resistance. Therefore, developing new chemotherapeutic drugs or eliminating drug resistance by inducing ferroptosis may represent a feasible approach. Studies should also examine the types of cancers that are very sensitive to ferroptosis as well as the genes/proteins that are up- and down-regulated.

Ferroptosis has been observed in several diseases with many genes involved as above. At this point, it is important to establish the optimal doses of ferroptosis inducers in order to reduce side effects. Ferroptosis can not only cure tumors, but also cause untoward effects in body. Ferroptosis cannot be experimentally induced by simply removing antioxidants from cells; therefore, ferroptosis is by no means a simple Fenton reaction. However, ferroptosis, as an important process, may offer new treatment options as our understanding of the process increases.

## Data Availability

Not applicable.

## Abbreviations

ROS: Reactive oxygen species
GPX4: Glutathione peroxidase 4
VDAC2/3: Voltage-dependent anion channels 2/3
HSP: Heat shock protein
NRF2: Nuclear factor erythroid 2-related factor 2
NADPH: Nicotinamide adenine dinucleotide phosphate
NOX: Nicotinamide adenine dinucleotide phosphate oxidase
GSH: Glutathione
TFR: Transferrin receptor 1
Glu: Glutamic acid
DLBCL: Diffuse large B cell lymphoma
Rb: Retinoblastoma protein
LncRNA: Long non-coding RNA
SCD1: Stearoyl-CoA desaturase 1
GRP78: Glucose-regulated protein 78
ATF4: Activator of transcription factor 4
